# *De Novo* or inherited: gonosomal mosaicism in hereditary angioedema due to C1 inhibitor deficiency

**DOI:** 10.3389/fimmu.2025.1550380

**Published:** 2025-02-06

**Authors:** Laura Batlle-Masó, Janire Perurena-Prieto, Laura Viñas-Giménez, Aina Aguiló-Cucurull, Paula Fernández-Álvarez, Johana Gil-Serrano, Mar Guilarte, Roger Colobran

**Affiliations:** ^1^ Infection and Immunity in Pediatric Patients Group, Vall d’Hebron Research Institute (VHIR), Barcelona, Catalonia, Spain; ^2^ Pediatric Infectious Diseases and Immunodeficiencies Unit, Hospital Universitari Vall d’Hebron (HUVH), Barcelona, Catalonia, Spain; ^3^ Pompeu Fabra University (UPF), Barcelona, Catalonia, Spain; ^4^ Translational Immunology Group, Vall d’Hebron Research Institute (VHIR), Barcelona, Catalonia, Spain; ^5^ Immunology Division, Vall d’Hebron University Hospital (HUVH), Barcelona, Catalonia, Spain; ^6^ Department of Cell Biology, Physiology and Immunology, Autonomous University of Barcelona (UAB), Bellaterra, Catalonia, Spain; ^7^ Department of Clinical and Molecular Genetics, Vall d’Hebron University Hospital (HUVH), Barcelona, Catalonia, Spain; ^8^ Department of Allergy, Vall d’Hebron University Hospital (HUVH), Barcelona, Catalonia, Spain; ^9^ Allergy Research Unit, Vall d’Hebron Research Institute (VHIR), Barcelona, Catalonia, Spain; ^10^ Department of Medicine, Autonomous University of Barcelona (UAB), Bellaterra, Catalonia, Spain

**Keywords:** hereditary angioedema, C1 inhibitor deficiency, SERPING1, somatic variant, gonosomal mosaicism, genetic counseling

## Abstract

Hereditary angioedema (HAE) is a rare genetic disease, characterized by transient and self-limiting episodes of subcutaneous or submucosal swelling that spontaneously resolve within two to five days. The most common form of HAE, HAE-C1-INH, is caused by deleterious mutations in the *SERPING1* gene, encoding the C1-Inhibitor protein, and its diagnosis is confirmed by decreased C1-INH function. Distinctively from other genetic forms of HAE, up to 15-20% of HAE-C1-INH cases are sporadic caused by *de novo* mutations. Here, we report a patient with apparently sporadic HAE-C1-INH. The patient had compatible clinical symptoms and a markedly low C1-INH function, and the parents showed normal values of C4 and normal C1-INH function. In the patient, we identified a novel splice site mutation in *SERPING1* (c.890-1G>C) and, by cDNA analysis, we confirmed its pathogenicity. Despite normal C1-INH function in the parents, we found that the mother was, unexpectedly, a mutation carrier. The inverted profile of the Sanger peaks compared with the patient, strongly suggested the presence of gonosomal mosaicism in the mother. We confirmed and quantified the mosaicism in different tissues by high depth NGS-based deep amplicon sequencing, showing a similar frequency of the variant ranging from 17 to 23%. In this study, we present the first case of gonosomal mosaicism in a family with a single child affected with HAE-C1-INH from unaffected parents. Our results underscore the importance of parental genetic testing in all patients, regardless of whether the parents are affected, and highlights the implications of gonosomal mosaicism for genetic counseling.

## 1 Introduction

Hereditary angioedema (HAE) is a rare genetic disease, with an estimated global prevalence of 1 in 50,000 to 1 in 100,000 individuals (1). HAE is characterized by transient and self-limiting episodes of subcutaneous or submucosal swelling that spontaneously resolve within two to five days. These episodes can occur in various areas, including the extremities, face, lips, tongue, gut and upper airways, the latter posing a life-threatening risk due to potential asphyxia. The clinical expression is highly variable, from asymptomatic individuals to patients suffering from frequent, unpredictable, disabling and potentially life-threatening attacks that severely affect their quality of life and that of their families (2). The pathophysiology of HAE involves dysregulation of endothelial permeability, leading to excessive plasma leakage from the blood vessels into surrounding tissues, resulting in transient swelling. The mechanisms underlying this dysregulation are heterogeneous, ranging from excessive production or action of bradykinin, a key mediator in inflammation and the regulation of blood pressure, to intrinsic abnormalities in specific components of the vascular endothelium (3).

The most common and classical form of HAE is caused by deleterious mutations in the *SERPING1* gene, encoding the C1-Inhibitor (C1-INH), a multifunctional plasma serine protease inhibitor involved in the regulatory network of complement, contact, coagulation, and fibrinolytic systems (4). Beyond this form of HAE with C1-INH deficiency, other genetic defects causing HAE have been described over time, most of them thanks to the use of high-throughput sequencing technologies (commonly known as next-generation sequencing, NGS). To date, eight different genes causing HAE in an autosomal dominant manner have been described: *SERPING1* (HAE-C1-INH) (5), *F12* (HAE-F12) (6), *PLG* (HAE-PLG) (7), *ANGPT1* (HAE-ANGPT1) (8), *KNG1* (HAE-KNG1) (9), *MYOF* (HAE-MYOF) (10), *HS3ST6* (HAE-HS3ST6) (11), *DAB2IP* (HAE-DAB2IP) (12); and, recently, *CPN1* has been reported as the first gene causing HAE in an autosomal recessive inheritance pattern (HAE-CPN1) (13). However, a significant number of patients with HAE and normal C1-INH do not carry pathogenic genetic variants in any of these genes. These cases are classified as HAE of unknown origin (HAE-UNK) (14).

HAE-C1-INH is the most prevalent and best-characterized form of HAE. In addition to clinical suspicion, the diagnosis of HAE-C1-INH is confirmed by the detection of low plasma levels of antigenic and/or functional C1-INH (usually accompanied by low C4 levels) (15). The last step in the diagnostic workflow is the genetic study. In the case of biochemically confirmed HAE-C1-INH, a genetic variant in the *SERPING1* gene should invariably be found. *SERPING1* is located in 11q12.1 and, despite being a small gene (contains eight exons and encodes a protein of 500 amino acids), more than 700 different variants scattered throughout the gene have been reported in patients with HAE-C1-INH (16). The most common types of these variants are missense and short deletions or duplications, but a very diverse spectrum of other genetic variants can be found in patients with HAE-C1-INH (nonsense, splice defects, large deletions and duplications, regulatory, etc.), acting by haploinsufficiency or by a dominant-negative mechanism (16–18).

The conventional approach for the molecular analysis of *SERPING1* includes the PCR amplification and direct sequencing (Sanger method) of all exons and the exon/intron flanking regions. With this method, the causing genetic variant can be found in up to 90% of patients (16). If no variant is found, the next step is to identify possible large gene rearrangements (e.g. deletions or duplications involving one or several exons) typically using multiplex ligation-dependent probe amplification (MLPA). These large gene rearrangements are particularly frequent in HAE-C1-INH (up to 10-15% of patients) because of the high incidence of DNA repetitive elements in *SERPING1* gene, which represent ‘hotspots’ for non-homologous recombination events (19). Finally, after Sanger sequencing and MLPA analysis, few patients (<5%) may remain genetically undiagnosed due to other types of genetic variants (promoter, deep-intronic, complex rearrangements, etc.), that can be challenging to identify and often require molecular methods that go beyond conventional approaches (20, 21). The use of NGS platforms that target the entire *SERPING1 locus* (including the promoter, exons, introns and 3’UTR) can overcome some limitations of the previous approaches (22). However, even with these advanced techniques, some genetic variants can remain unidentified and, importantly, their implementation in routine practice may not be feasible in many centers due to cost-effectiveness concerns.

Some characteristics of *SERPING1* are responsible for the higher than average mutation rates that this gene has: i) its location close to the centromere, as these genes tend to have a higher mutation rate due to a combination of factors related to chromosome structure, replication, and the maintenance of genetic material during cell division (23). ii) The high incidence of DNA repetitive elements (especially Alu sequences), which favors non-homologous recombination events that may cause partial deletions or duplications of the gene (24). iii) The high frequency of CpG sites that represent mutational ‘hotspots’ as they are prone to spontaneous deamination (19).

All these features also explain the high rate (up to 15-20%) of HAE-C1-INH cases with no family history of the disease (i.e. sporadic cases) that arise from *de novo* genetic variants in *SERPING1*. Deciphering whether a *SERPING1* variant in a patient is inherited (transmitted by the parents) or *de novo* (occurring for the first time in the patient) is crucial for understanding the possible familial implications of the disease and for adequate genetic counseling.

According to their origin, genetic variants can be divided into germline or somatic. A variant is considered germline when it is already present in the zygote and, thus, it is present in all cells of an individual. Germline variants can be originated *de novo*, or they can be inherited from either parent. In contrast, somatic variants are originated post-zygotically and are always *de novo*. Depending at which stage in the embryonic development (or in the postnatal life) the somatic variant appears, the percentage of cells or tissues affected will be different, leading to an individual with one of these three types of mosaicism: i) Germline mosaicism (also known as gonadal mosaicism) refers to a somatic variant that is only present in gonads (ovaries and testicles) and can cause apparently *de novo* variation in the next generation. ii) Somatic mosaicism, affecting cells other than germline cells. In this case, the variant will not be transmitted to the offspring. iii) Gonosomal mosaicism, a combination of germline and somatic mosaicism that refers to somatic variants present in gonads and other tissues. In the case of gonosomal mosaicism, the somatic variant can be transmitted to the next generation so it can also cause apparently *de novo* variation (25).

Here, we report an apparently sporadic case of HAE-C1-INH, which, after familial genetic testing, was found to be inherited from the asymptomatic mother carrying the *SERPING1* genetic variant in the form of gonosomal mosaicism. We detail how to address the molecular study of this phenomenon and we discuss the importance of familial genetic testing and the implications of gonosomal mosaicism for genetic counseling.

## 2 Methods

### 2.1 Subjects of the study

The reported family consists of three members, the index patient and both parents, who attended the Allergy Division of Vall d’Hebron University Hospital (Barcelona, Spain). Written informed consent for the studies reported here and for publication of the article was obtained from both parents, according to the procedures of the Clinical Research Ethics Committee of the Vall d’Hebron University Hospital [code: PR(AG)202/2021].

### 2.2 Laboratory tests

Serum levels of C3, C4, C1q and C1-INH were determined by turbidimetry (Optilite, Binding Site, UK). C1-INH functional activity was determined by ELISA (QuidelOrtho, USA).

### 2.3 *SERPING1* molecular screening

Genomic DNA was extracted from peripheral blood collected in EDTA tubes using a QIAamp DNA Mini Kit (Qiagen, Germany) according to the manufacturer’s instructions. The eight exons of *SERPING1* gene and their flanking regions were amplified by PCR (primers and PCR conditions are available upon request). Purified PCR products were sequenced by Sanger method using an Applied Biosystems 3500 Genetic Analyzer (Thermo Fisher Scientific, USA).

Since we identified a sequence substitution in a canonical splice site, we analyzed the potential alterations in RNA processing. Total blood was collected using Tempus™ Blood RNA Tubes (Thermo Fisher Scientific, USA) and total RNA was isolated using the Tempus™ Spin RNA Isolation Kit (Thermo Fisher Scientific, USA). cDNA was synthesized from 1 μg of RNA using anchored-oligo(dT)18 primers and with the Transcriptor First Strand cDNA Synthesis Kit (Roche, Switzerland).

The *SERPING1* cDNA region containing the c.890-1G>C genetic variant was amplified using primers located in exons 4 and 7 and purified PCR products were sequenced by Sanger method (primers and PCR conditions are available upon request).

### 2.4 Evaluation of gonosomal mosaicism

Samples from the patient’s mother were analyzed to determine the percentage of mosaicism in different specimens (peripheral blood, buccal swab and urine). Genomic DNA was extracted using a QIAamp DNA Mini Kit (Qiagen, Germany) according to the manufacturer’s instructions. PCR products encompassing the region of interest (c.890-1G>C variant) were used for Sanger sequencing and NGS-based deep amplicon sequencing (NGS-DAS) to precisely quantify the variant allele frequency (VAF). NGS-DAS was performed with the MiSeq platform (Illumina, USA). Briefly, PCR products were quantified with a Qubit 2.0 Fluorometer (Thermo Fisher Scientific, MA, USA). A 300- to 900-ng amount of DNA was fragmented using NEBNext dsDNA Fragmentase (New England Biolabs, Ipswich, MA, USA) to obtain fragments of approximately 200 bp in size. End repair, ligation of the adapter for Illumina sequencing, and sample indexing through a brief amplification were then performed using the NEBNext Ultra DNA library prep kit for Illumina and the NEBNext MultiplexOligos for Illumina Dual Index Primers Set 1 (New England Biolabs, Ipswich, MA, USA). All necessary purifications and size selections to recover only the fragments of interest were done using AMPure XP beads (Beckman Coulter). Libraries were quality-evaluated using QIAxcel (Qiagen) and quantified with a Qubit 2.0 Fluorometer. The DNA sample libraries were then mixed in equimolecular amounts and sequenced in a MiSeq instrument (Illumina, San Diego, CA, USA) using the 500-cycle MiSeq reagent kit v2 with a paired-end run of 2×250-bp reads. All procedures were performed according to the manufacturer’s instructions.

### 2.5 Bioinformatics analysis

NGS sequencing data were aligned to the reference genome, hg38, using BWA (https://github.com/lh3/bwa). Duplicate reads were marked using Picard MarkDuplicates (version 2.18.6, https://github.com/broadinstitute/picard). Next, GATK 4.1.8.1 (https://github.com/broadinstitute/gatk) was used to calibrate the data, calculate BQSR scores (BaseRecalibrator, ApplyBQSR), and perform variant calling (Mutect2). We then annotated the resulting VCF files using Annovar (https://annovar.openbioinformatics.org/en/latest/). Finally, we used Integrative Genomic Viewer to count the number of reads supporting each allele.

## 3 Results

### 3.1 Clinical description of a patient with apparently sporadic hereditary angioedema due to C1-INH deficiency

A 13-year-old girl was evaluated in the Allergy Department of the Vall d’Hebron University Hospital. Since the age of three, the patient had experienced recurrent episodes of severe, colicky abdominal pain accompanied by nausea and vomiting, lasting 3-4 days. These episodes frequently required hospitalization for intravenous fluids, antiemetics, and parenteral analgesics. Extensive evaluations, including tests for celiac disease, inflammatory bowel disease, and irritable bowel syndrome, were inconclusive. She was initially misdiagnosed with cyclic vomiting syndrome. At age five, the patient developed edema in the genital and perineal regions following a bicycle-related trauma. Subsequently, she experienced multiple peripheral angioedema attacks involving the hands, feet, and lips, often triggered by minor trauma. Episodes were unresponsive to antihistamines and corticosteroids but typically resolved within 2–3 days. Some attacks were preceded by erythema marginatum.

Laboratory evaluation revealed low C4 and C1-INH antigenic levels and a markedly low C1-INH function (**Table 1**), pointing to a diagnosis of type I HAE-C1-INH. The patient had no siblings, and her parents reported no history of angioedema. Laboratory evaluation of the parents showed normal values of C4, C1-INH and normal function of C1-INH (**Table 1**), suggesting that the patient was a sporadic case.

**Table 1:** Laboratory findings across family members.

Parameter	Patient	Father	Mother	Reference Values
Complement C3 (mg/dL)	97.3	108	139	85-180
Complement C4 (mg/dL)	**↓ 3.57**	24.6	20	10-40
Component C1q (mg/dL)	23.2	21.4	28.5	16-31
C1 inhibitor (mg/dL)	**↓ <8.5**	38	24.6	21-38
C1 inhibitor (% function)	**↓ 37%**	100	83	>67%

Values that deviate from the reference values are shown in bold.

↓, value is decreased compared to the reference values.

At diagnosis, the patient experienced an average of 2-3 attacks per month. Her baseline scores were as follows: Angioedema Control Test (AECT): 6 (range 0-16; <10 poorly controlled) (26), Hereditary Angioedema Activity Score (HAE-AS): 12 (range 0-19) (27), and Angioedema Quality of Life Questionnaire (AE-QoL): 40 (range 0-68; a higher score means worse QoL) (28). Acute attacks were managed effectively with subcutaneous icatibant acetate (30 mg). For long-term prophylaxis, treatment with berotralstat (150 mg/day) was initiated.

### 3.2 Identification of a novel *SERPING1* pathogenic variant leading to aberrant splicing

**Figure 1:**
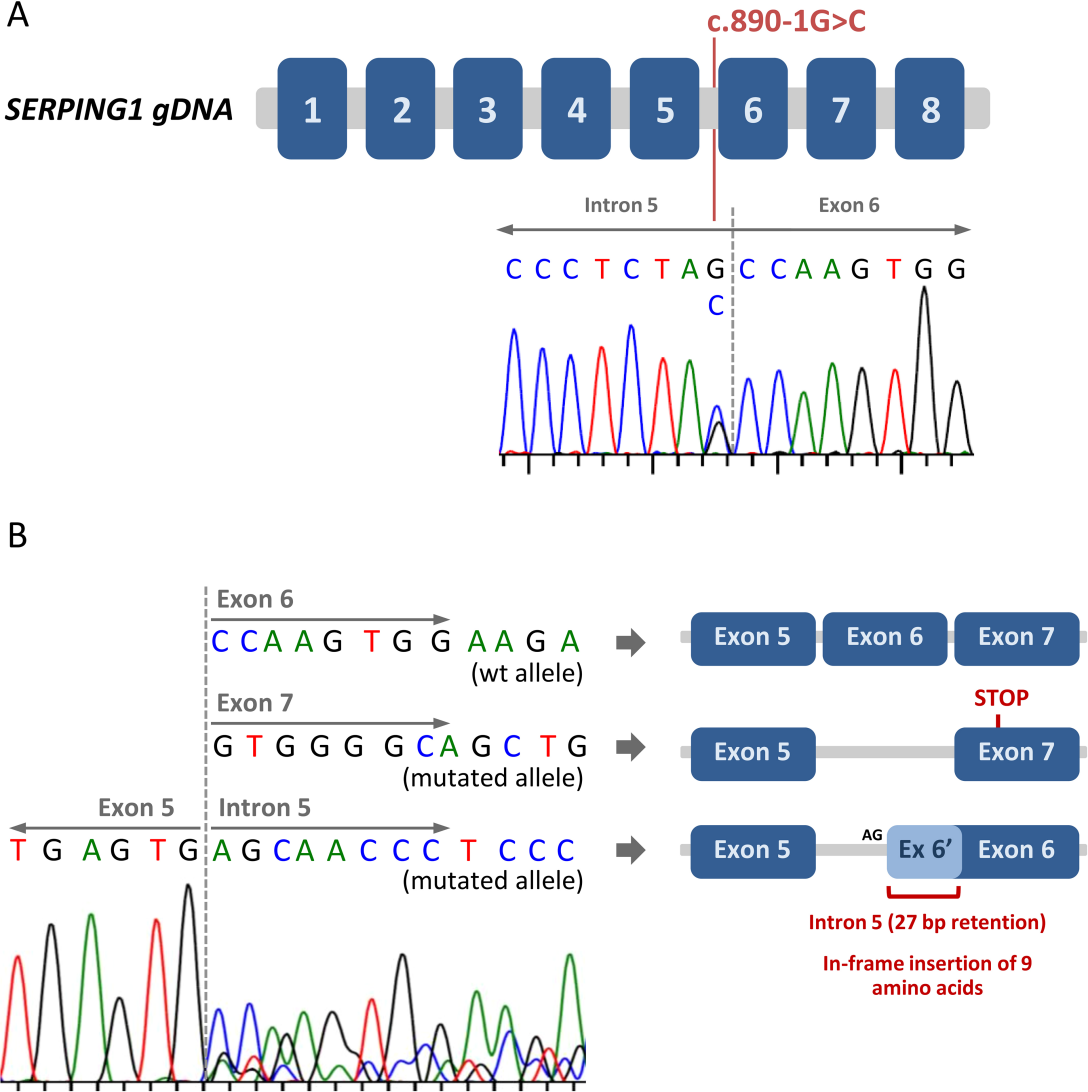
Identification of a novel splice site mutation in *SERPING1*. **(A)** Schematic representation of the *SERPING1* gene. The eight exons are represented by blue boxes. The Sanger chromatogram corresponding to the index patient is shown, and the c.890-1G>C genetic variant (located at the end of intron 5) is indicated. **(B)** The consequences of the *SERPING1* c.890-1G>C genetic variant on gene transcription and splicing were assessed by mRNA analysis. After exon 5, three different sequences were detected, corresponding to the wild type allele and two different aberrantly spliced mRNAs from the mutated allele. A schematic view of the different resulting transcripts is included.

Given the clear evidence of type I HAE-C1-INH in the patient, we proceeded with the sequencing of the *SERPING1* gene. We sequenced all exons and flanking regions (at least 20 nucleotides at each end) using the Sanger method. We found a heterozygous genetic variant affecting the essential acceptor splice site of intron 5, NM_000062.3:c.890-1G>C (**Figure 1A**). This variant was novel, although a different nucleotide change in the same position (c.890-1G>A) had been previously described in a patient with type I HAE-C1-INH without demonstrated evidence of a splicing defect (29). The mutation found in our patient (c.890-1G>C) was not present in population databases (gnomad v4.1.0) and, according to the in-silico predictor SpliceAI (30), it was predicted to disrupt the acceptor splice site of intron 5 (SpliceAI score: 0.98).

To accurately determine the effect of the c.890-1G>C mutation in the mRNA of *SERPING1*, we obtained mRNA from the patient’s total blood, generated the cDNA and amplified and sequenced a region from exon 4 to exon 7. The analysis of the sequence showed that the mutated allele produced two different aberrantly spliced transcripts: i) Transcripts with exon 6 skipping. In this case, the new junction between exon 5 and 7 produced a frameshift leading to a premature STOP codon in the first half of exon 7 (**Figure 1B**). The predicted truncated protein would have only 320 amino acids (compared with the 500 amino acids of the wild type C1-INH). ii) Transcripts with a partial intron 5 retention. Here, the loss of the canonical acceptor splice site (the AG dinucleotide located at positions -1 and -2 relative to exon 6) activate a cryptic acceptor splice site located in intron 5 (positions -28 and -29 with respect to exon 6). The use of this alternative acceptor splice site includes 27 nucleotides of the intron 5 in the novel exon 6 (**Figure 1B**). This means that, at a protein level, there is a predicted in-frame insertion of 9 amino acids.

In summary, the c.890-1G>C mutation clearly alters the splicing process of *SERPING1*, causing two different aberrant splicing phenomena (exon 6 skipping and partial intron 5 retention). According to the size of the peaks in the Sanger chromatogram, both types of aberrant transcript are produced in the same ratio (**Figure 1B**). Based on all the evidence, we classified this variant as pathogenic, reported it to ClinVar (accession number: SCV005439126) and considered it causative of the patient’s type I HAE-C1-INH.

### 3.3 Identification of a gonosomal mosaicism for the *SERPING1* c.890-1G>C mutation in the patient’s mother

**Figure 2:**
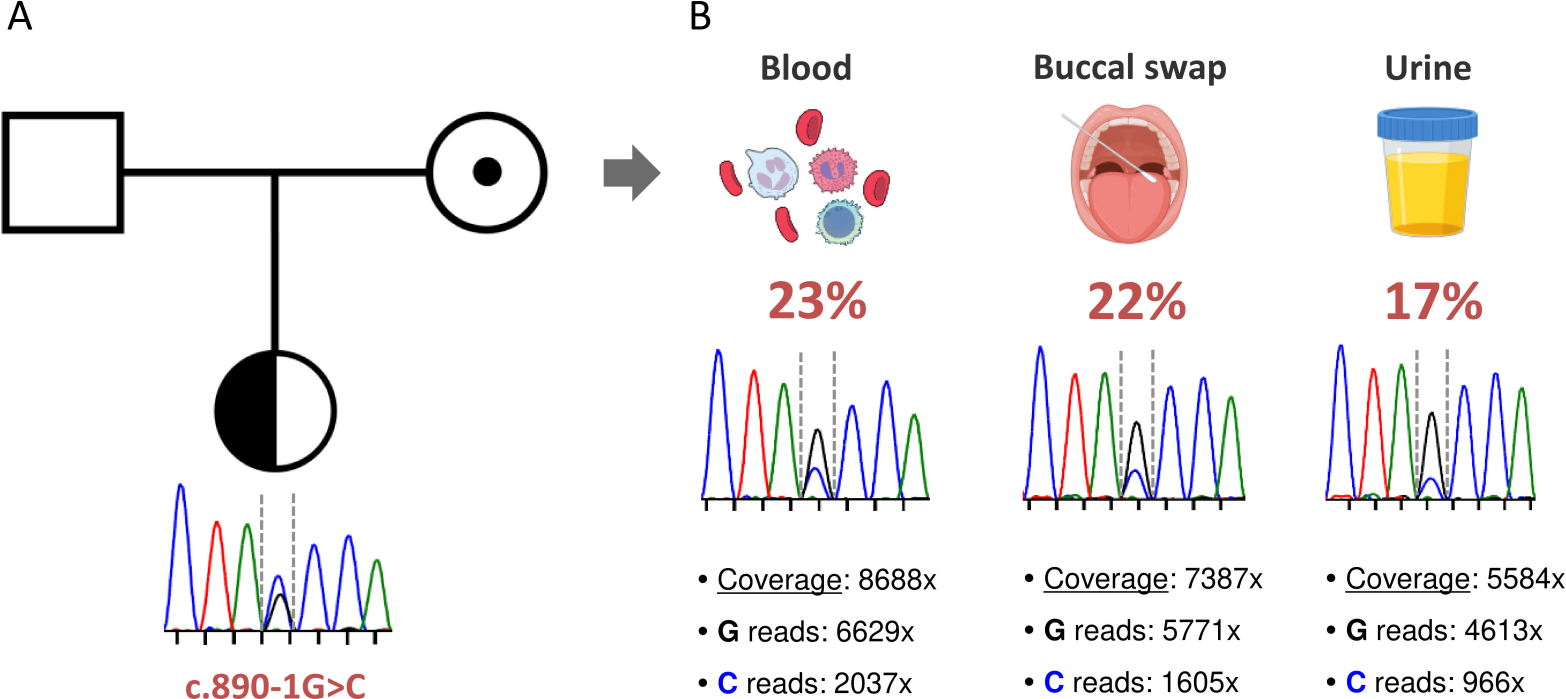
Identification of a maternal gonosomal mosaicism. **(A)** Pedigree and familial segregation of the *SERPING1* c.890-1G>C genetic variant are shown. The index patient is heterozygous (represented with a half-black circle), and the mother carries the genetic variant in the form of gonosomal mosaicism (indicated by a black dot). **(B)** The presence of the *SERPING1* c.890-1G>C genetic variant was assessed in different tissues from the mother. For each tissue, the Sanger chromatogram and the results of NGS-based deep amplicon sequencing are shown. Coverage refers to the total number of sequences that include the position of the genetic variant of interest. The specific number of reads containing each allele and the frequency of the somatic variant in each tissue are also specified.

The laboratory tests performed on the patient and her parents indicated that this was a sporadic case of HAE-C1-INH, since both parents exhibited normal values of C4, C1-INH and normal function of C1-INH. Despite this, we decided to proceed with the genetic study of both parents to complete the genetic evaluation of the family. Unexpectedly, we found that the mother was a carrier of the c.890-1G>C mutation (**Figure 2A**). A careful examination of the Sanger peaks showed that there was an inverted profile of the two alleles in the mother compared with the patient (**Figure 2B**, blood sample). Specifically, the size of the peak corresponding to the mutated allele was significantly lower than that of the wild type. To rule out any bias causing selective allele amplification, we designed a second pair of primers to amplify and sequence the region of the c.890-1G>C mutation. The results were exactly the same (data not shown), strongly suggesting the presence of gonosomal mosaicism in the mother.

To precisely quantify the variant allele frequency (VAF) of the mutation in the mother, we performed both Sanger sequencing and NGS-based deep amplicon sequencing (NGS-DAS) of the c.890-1G>C mutation using DNA from hematological and non-hematological samples. We obtained high on-target depth coverage (from 5,500x to 8,600x), and we detected the *SERPING1* c.890-1G>C mutation in all samples analyzed at similar frequencies: blood (23%), buccal swab (22%) and urine (17%) (**Figure 2B**). All these findings confirm that the patient’s mother, although clinically asymptomatic and showing no alterations in C4 and C1-INH levels, is a carrier of the *SERPING1* c.890-1G>C mutation in the form of gonosomal mosaicism.

## 4 Discussion

HAE-C1-INH is the most common type of HAE and its diagnosis is based mainly on clinical symptoms, a positive family history (although this may not be present in up to 15-20% of patients), and, finally, laboratory tests, with measurements of plasma levels of C4, C1-INH protein and C1-INH function being used to diagnose HAE-C1-INH with high accuracy (15). Counterintuitively, despite the genetic nature of HAE-C1-INH, the disease is typically diagnosed based on clinical and biochemical findings (31). According to the last update of the international WAO/EAACI guideline for the management of HAE “Sequencing of the *SERPING1* gene can be supportive in the diagnostic workup of some HAE-C1-INH (including prenatal diagnosis); however, biochemical C1-INH testing is effective and less expensive than genetic” (15). Therefore, genetic testing is not mandatory for HAE-C1-INH diagnosis. However, genetic testing can be very relevant in certain cases, such as when there is suspicion of gonadal mosaicism, as in the case presented in our study. The identification of mosaicism can have important implications for genetic counseling, helping to clarify the hereditary risk for future offspring, especially when parents are unaware of the possibility of transmitting the disorder. Providing families with information about the likelihood of recurrence, the possibility of prenatal testing, and the implications for other family members is crucial for informed decision making. In addition, genetic counseling can offer emotional support, address any family planning concerns, and guide the patient and family through the treatment strategies available for HAE.

Here, we studied a family with a patient suffering from HAE-C1-INH due to a novel *SERPING1* mutation. Both parents were asymptomatic and presented normal values of C4, C1-INH protein and function, indicating that the patient had a sporadic HAE-C1-INH. However, after familial genetic testing, we found that the mother was a carrier of the *SERPING1* mutation in the form of gonosomal mosaicism.

Several pieces of evidence led us to suspect gonosomal mosaicism in the patient’s mother in this study. First, the mother was an asymptomatic carrier of the pathogenic *SERPING1* variant. This fact alone is not enough to suspect the presence of a somatic variant since disease penetrance in HAE-C1-INH is very high but incomplete. Second, the mother showed normal values of C4, C1-INH protein and normal function of C1-INH. This was unexpected because pathogenic variants in *SERPING1* gene invariably lead to low C1-INH function, regardless of whether or not the disease manifests and independently of the degree of severity. Third, when analyzing the mutation’s position in the Sanger chromatogram, the mother showed an inverted pattern of the peaks corresponding to the wild type and mutated alleles compared with the patient. Specifically, the mother had a significantly smaller peak corresponding to the mutated allele. In such cases, repeating amplification and sequencing with at least one different set of primers is highly recommended to exclude that the smaller peak resulted from a bias during the PCR. We verified this inverted pattern with an additional primer pair, thus confirming that the mother carried a somatic pathogenic variant (c.890-1G>C) in *SERPING1*. The fact that the mother transmitted the variant to her daughter led to the conclusion that this somatic variant was in the form of gonosomal mosaicism. Besides the presence of the variant in gonads and blood, we also identified the c.890-1G>C variant in non-hematopoietic tissues through the study of DNA from buccal swab and urine. By NGS-DAS we precisely quantified the VAF, which was similar in all analyzed samples (between 17% and 23%). Although we could not study the degree of mosaicism in the liver (where C1-INH is mainly produced), it is reasonable to believe that it will not differ significantly from that obtained in the different analyzed samples. Hypothetically, if we assume a VAF of 20% in the liver, it means that four cells out of ten (40%) will carry the heterozygous mutation. Therefore, there will be 60% of wild-type cells that can produce a normal amount of C1-INH, and 40% of cells that carry the mutation and produce reduced amounts of C1-INH. The fact that the mother has normal quantity and function of C1-INH suggests that an individual with approximately 60% of wild type cells can produce enough C1-INH to be biochemically normal and clinically healthy.

In this study, we used both Sanger sequencing and NGS technologies. To initially discover the variant in the patient, we used Sanger sequencing, as it is the routine diagnostic method for HAE at our center. In this case, the variant could also have been identified using NGS, as the allele frequency of the variant in the patient (heterozygous, VAF ~50%) would have been detected by standard variant calling pipelines. In the case of the mother, different technologies may have led to different outcomes. Using Sanger sequencing, the method we typically use for family segregation, we easily detected the variant in the mother and realized that there was an allelic imbalance. However, if NGS had been used, it might have been more difficult to identify the somatic variant, as many NGS pipelines filter out variants that deviate from typical heterozygous or homozygous calls. That is why we used a somatic variant caller (Mutect2) to analyze the NGS data from the different maternal samples. Once the somatic variant is correctly identified, NGS has a clear advantage over Sanger: absolute and accurate quantification of both alleles. This study exemplifies how Sanger and NGS can be combined for the detection and quantification of germline and somatic variants.

Gonosomal mosaicism is due to a mutational event during early embryogenesis and, consequently, the genetic variant is present in a portion of both somatic and gonadal cells (32). This means that the affected individual may pass the mutation to the offspring, which has clear implications on genetic counseling, especially when compared with the scenario of a truly *de novo* mutation. Parents of HAE-C1-INH patients who are carriers of germline *SERPING1* mutations have a recurrence risk of 50%, whereas patients with true *de novo* mutations have a recurrence risk similar to that of the general population (negligible in terms of genetic counseling). In our patient’s mother, the somatic mutation was detected at a similar frequency in all samples analyzed but, although tempting, we cannot assume that this frequency will be the same in gonads. Therefore, a precise assessment of recurrence risk is difficult in the case reported here. Adopting a conservative approach, we should assume the same risk as in germline *SERPING1* mutations (50%) by default (33).

To date, few cases of mosaicism in HAE-C1-INH have been reported. In 2006, Guarino et al. reported gonadal mosaicism in a family with two affected siblings carrying a nonsense mutation in *SERPING1*. Neither of the parents carried the mutation in blood or any other tissues, pointing to mosaicism restricted to the gonads (34). A second case of gonadal mosaicism was reported in 2018 by Didier G Ebo et al. As in the previous study, the suspicion of mosaicism arose when they found two siblings with the same *SERPING1* mutation and neither parent was a carrier. In this case, the authors demonstrated paternal gonadal mosaicism by analyzing DNA from the father’s sperm (35). The only case of gonosomal mosaicism was published in 2007 by Tai-Chang Yu and collaborators, in a family with two symptomatic siblings. Both parents were asymptomatic but the father carried the *SERPING1* mutation in blood and buccal cells at a ratio that indicated the presence of gonosomal mosaicism (36). All of these previously reported cases have in common a high suspicion of mosaicism, as an autosomal dominant disorder (HAE-C1-INH) occurred in more than one child from unaffected parents. In these cases, clinicians should consider the possibility of mosaicism (gonadal or gonosomal) in one of the parents, particularly after ruling out nonbiological parenthood.

Here, we reported the first case of gonosomal mosaicism in a family with a single child affected with HAE-C1-INH from unaffected parents. In this situation, the vast majority of cases are due to *de novo* mutations, especially because *de novo* mutations are particularly frequent in HAE-C1-INH (19). Moreover, both parents were not only asymptomatic, but also showed completely normal values of C4, C1-INH protein and function. Usually this is enough to assume that the patient is a sporadic case due to a *de novo* mutation and, in most cases, the genetic testing of the parents is not performed. Our study emphasizes the importance of parental genetic testing in all patients, regardless of whether the parents are affected or not. This is especially crucial in families with a desire to have more children, since genetic counseling changes drastically depending on whether the mutation arises *de novo* in the patient or is inherited as a result of mosaicism in a parent.

## Data Availability

The datasets presented in this study can be found in online repositories. The names of the repository/repositories and accession number(s) can be found in the article/supplementary material.
